# Global optimization of extraframework ensembles in zeolites: structural analysis of extraframework aluminum species in MOR and MFI zeolites†

**DOI:** 10.1039/d2cp03603g

**Published:** 2022-10-11

**Authors:** Elena V. Khramenkova, Harshini Venkatraman, Victor Soethout, Evgeny A. Pidko

**Affiliations:** Inorganic Systems Engineering group, Department of Chemical Engineering, Faculty of Applied Sciences, Delft University of Technology, Van der Maasweg 9 2629 HZ Delft The Netherlands E.A.Pidko@tudelft.nl

## Abstract

Metal-modified zeolites are versatile catalytic materials with a wide range of industrial applications. Their catalytic behaviour is determined by the nature of externally introduced cationic species, *i.e.*, its geometry, chemical composition, and location within the zeolite pores. Superior catalyst designs can be unlocked by understanding the confinement effect and spatial limitations of the zeolite framework and its influence on the geometry and location of such cationic active sites. In this study, we employ the genetic algorithm (GA) global optimization method to investigate extraframework aluminum species and their structural variations in different zeolite matrices. We focus on extraframework aluminum (EFAl) as a model system because it greatly influences the product selectivity and catalytic stability in several zeolite catalyzed processes. Specifically, the GA was used to investigate the configurational possibilities of EFAl within the mordenite (MOR) and ZSM-5 frameworks. The *x*TB semi-empirical method within the GA was employed for an automated sampling of the EFAl–zeolite space. Furthermore, geometry refinement at the density functional theory (DFT) level of theory allowed us to improve the most stable configurations obtained from the GA and elaborate on the limitations of the *x*TB method. A subsequent *ab initio* thermodynamics analysis (aiTA) was chosen to predict the most favourable EFAl structure(s) under the catalytically relevant *operando* conditions.

## Abstract 2

An expert bias-free *operando* computational modelling study of the structure and stability of extraframework aluminum species in the pores of mordenite and ZSM-5 zeolites is presented.

## Graphical abstract

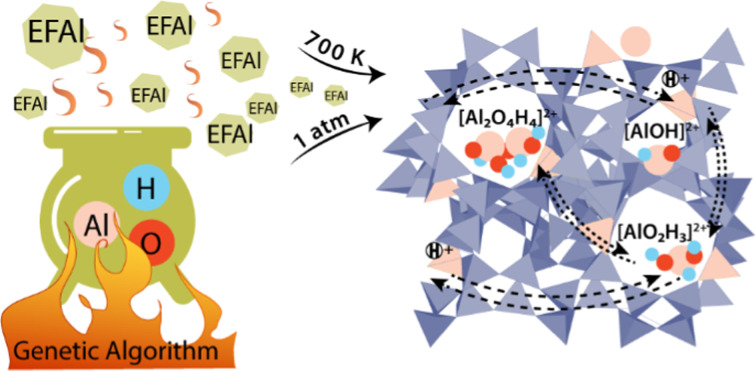

## 1. Introduction

The development of advanced predictive models in heterogeneous catalysis requires a deep insight into the molecular-level structure of the active sites, their catalytic mechanism and evolution under operating conditions.^1,2^ The structural complexity and heterogeneity of common solid catalysts are major challenges in constructing representative active site models, which are conventionally performed using a combination of various indirect characterization data and chemical intuition of researchers.^3^ Global optimization techniques provide a practical approach to reducing the expert bias in addressing the active site structure in heterogeneous catalysts.^4^

One of the most popular global optimization (GO) techniques is the genetic algorithm (GA) which allows scanning of the potential energy surface (PES) of a given system and prediction of the most stable structures *via* an evolutionary algorithm.^3^ The underlying principles of the algorithm are based on learning the “good” features of the possible structural moieties and improving them throughout the alterations of continuous structures. In this manner, a pool of advanced solutions can be identified which is otherwise challenging to guess based on chemical intuition and spectroscopic data only.^5,6^ GO algorithms have been successfully used to address the structural problem of the active sites in heterogeneous catalysis including oxides,^7,8^ supported metal nanoparticles,^9^ electrocatalytic interfaces,^10^ and, since quite recently, also zeolite-based catalysts.^11,12^

Zeolites are porous, crystalline aluminosilicates, whose reactivity and catalytic behaviour can be widely adjusted by modifying them with extraframework (EF) metal-containing species.^13,14^ The structural and catalytic properties of such EF species depend on the chemical composition (lattice Si/Al ratio), the presence of heteroatoms, and the topological properties of the confining zeolite matrix.^15^ As the spectrum of plausible variations in features is large, it is important to understand the role of zeolite topology and the nature of the extraframework aluminum (EFAl) species, which largely influence the activity of zeolites.

The extraframework aluminum species situated in zeolites hold an important position in zeolite chemistry and catalysis. The formation of Lewis acidic EFAl in the zeolite pores during the synthesis or post-synthetic activation modulates the acidity, catalytic reactivity, and long-term stability of zeolite-based catalysts.^16,17^ The formation of EFAl often results in releasing aluminum from the framework in procedures of high-temperature steaming, calcination or acid/base leaching.^18^ The structures of the EFAl are not completely known; however, some authors quote the condensed and uncondensed species. The former are the phases of Al_2_O_3_, which are formed at the external surfaces of zeolites, while the latter are aluminum and oxoaluminum cations such as AlO^+^, AlOH^2+^, and Al^3+^.^19^ The formation of these mononuclear species has been proposed based on the results of NMR spectroscopy and DFT calculations, which, until recently, have been mostly considering small mononuclear EF aluminum.^16,20^ It has also been demonstrated that the strength of the BAS can be enhanced in the presence of AlOH^2+^. However, DFT calculations combined with *ab initio* thermodynamic analysis (aiTA) have shown that, under the conditions of zeolite activation, the formation of the multinuclear Al_3_O_4_H_3_^4+^ species inside FAU can be observed.^16^ A driving force for the formation of the EFAl complexes with high nuclearity was stated to be the high basicity of terminal O-containing groups and the unsaturation of Al centers in these complexes. The experimental observation of the multinuclear species was demonstrated by Zheng *et al.* using the combined ^31^P solid-state NMR and 1,2-bis(dimethylphosphine) ethane probe molecule method.^21^

EFAl species are speculated to reduce the effective pore size within the zeolitic framework leading to better stabilization of intermediates and transition states during the catalytic process.^22^ Another proposal states that EFAl species polarize the BAS in their vicinity which in turn increases the reactivity of the BAS.^23,24^ Although several insights were gained through earlier spectroscopic and theoretical studies of EFAl moieties, their exact geometry still remains elusive. The spectroscopic characterization of EFAl yields a general understanding of the coordination environment but provides limited information on the structural changes induced by the reactive conditions which are essential to discern the catalytic role of these species.^3^

**Scheme 1:**
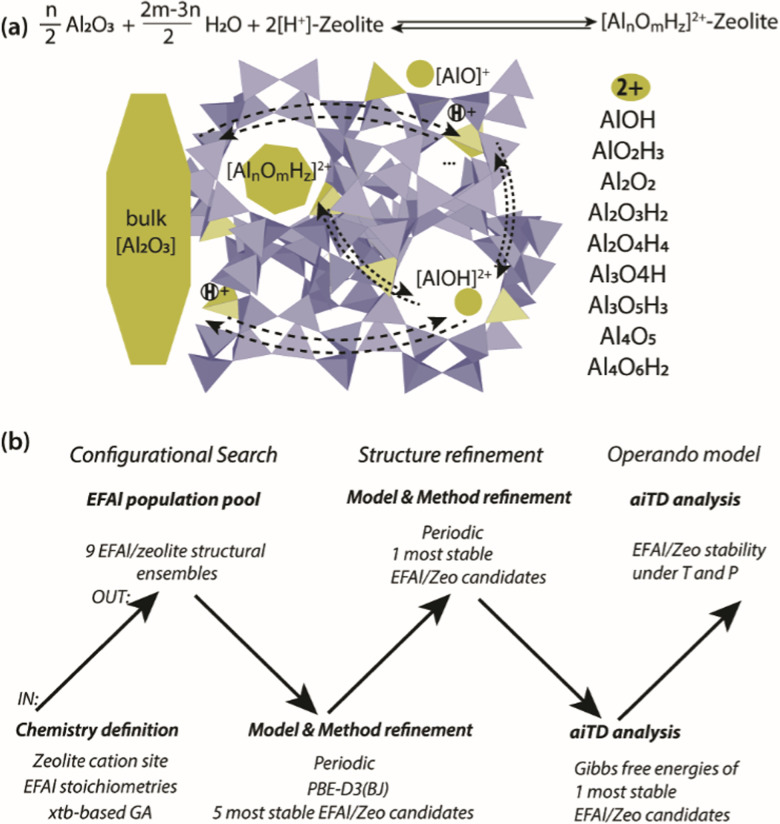
(a) The model definition for the interconversion of EFAl species in a zeolite matrix with a list of stoichiometries included in the current stability analysis of molecular intrazeolite EFAl with a reference to separate phases of bulk Al_2_O_3_ and the acidic zeolite matrix (2[H^+^]–zeolite). The structure prediction and stability evaluation of the EFAl–zeolites followed a hierarchical approach (b) involving the initial configurational search using a semi-empirical (*x*TB)-based configurational search followed by structural refinement using a full periodic DFT modelling and subsequent aiTA to evaluate the stability of the obtained extraframework ensembles under the catalytically relevant conditions.

Herein, we present a computational study aimed at addressing the EFAl structures in zeolites (Scheme 1(a)). The computational workflow implemented to achieve this is schematically illustrated in Scheme 1(b). For a given selection of EFAl stoichiometries encapsulated in MOR or ZSM-5 zeolites, an exhaustive scanning of the PES was carried out using the GA based on *x*TB semi-empirical calculations. The structures produced at the end of the GA configurational search were further refined by fully periodic DFT calculations. Subsequently, the stabilities of the structurally refined stoichiometries under catalytically relevant conditions were evaluated in the framework of aiTA. The workflow implemented in this study provides a bias-free approach for the structural analysis of extraframework species in zeolites and allows configurations to be found that could not be previously anticipated.

## 2. Computational methods

### Configurational search

**Fig. 1:**
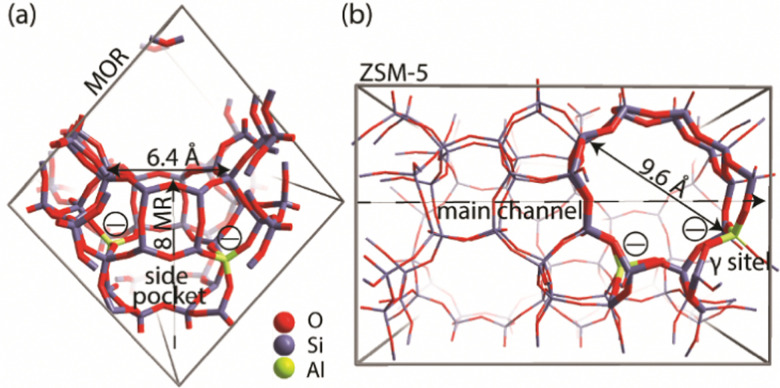
(a) Location of the cluster model utilized in the genetic algorithm for the global minima search of cationic sites incorporated in the periodic model of the mordenite. (b) Location of the cluster model utilized in the genetic algorithm for the global minima search of cationic sites incorporated in the periodic model of the ZSM.

A genetic algorithm (GA) was used to exhaustively explore the configurational space formed by the isomeric structures of 9 different EFAl stoichiometries, individually. Fig. 1 presents the cluster models of the zeolites utilized in the GA search, which are highlighted as a part of their respective periodic structures. Such structural models were used earlier as they resemble the model catalysts of EFAl-containing zeolites prepared by impregnation and ion exchange processes. By selecting a side pocket of MOR and γ-site of ZSM-5 as the cation sites accommodating EFAl, a uniform confinement effect was created across EFAl in respective frameworks.^25,26^ The models assumed the defect-free zeolite lattice and uniform confinement environment to facilitate analysis and structural exploration. The detailed information on the pore size and zeolitic topologies is present in Section S.1. of the ESI.† The choice of the EFAl stoichiometries considered in this study (Scheme 1(a)) was inspired by evidence from previous findings on the formation of mononuclear species such as AlOH^2+^ and AlO_2_H_3_^2+^, binuclear species such as Al_2_O_2_^2+^, Al_2_O_3_H_2_^2+^, and Al_2_O_4_H_4_^2+^, and the spontaneous formation of multinuclear EF aluminum such as Al_3_O_4_H^2+^, Al_3_O_5_H_3_^2+^, Al_4_O_5_^2+^ and Al_4_O_6_H_2_^2+^.^20,27–30^ Note that these stoichiometries correspond to complete ensembles. These ensembles within zeolites can be represented as a single species with a +2 overall charge and by a combination of species having the same total charge (*e.g.* a +1 EFAl cation and a zeolite BAS or a neural EFAl and two BAS). The cationic charge of the EF ensemble was compensated by two negatively charged AlO_2_^−^ lattice units.

The configuration exploration was carried out using the version of the GA developed by Vilhelmsen and Hammer.^31^ The operations of the fitness assignment, crossover, mutation, and selection were executed in the GA using the atomic simulation environment (ASE) with the semi-empirical tight-binding calculator GFN1 – *x*TB.^31–34^ The mutation probability of the GA was chosen as 30%, and the population size at the end of each generation was set to consist of 20 structures. During the GA search, only the positions of the extraframework species were allowed to change, while the coordinates of the cluster model atoms were kept frozen. The parameters of the maximum energy difference, the maximum difference in the interatomic difference and the maximum interatomic distances were set to 0.02 eV, 0.7 Å, and 0.015 Å, respectively. The convergence was assumed to be reached when the last 5 consecutive generations of the population pool were identical.

### Structure refinement

Since the GFN-*x*TB methods proved to be reliable for the geometries and frequencies calculations, but not the energies, GFN1-*x*TB optimized configurations were further refined at the DFT level using more realistic periodic zeolite models. The structural refinement was carried out at the PBE-D3(BJ) level of theory.^35^ The final pool of each GA run was examined and five lowest-energy configurations were extracted for the structural refinement. The compromise choice of 5 lowest lying candidates is motivated by our preliminary cluster calculations on mononuclear EFAl species in MOR (see Section S.2. in the ESI†), showing that such a choice allows the minimum-energy considerations to be found, whilst providing a good compromise between the accuracy and computational costs. For the structure refinement using periodic DFT (pDFT) calculations, the configurations obtained with the *x*TB GA on cluster models were directly transferred into the respective positions of the fully periodic zeolite models.

pDFT calculations were carried out using the Vienna *Ab Initio* Simulation Package (VASP)^36^ with the generalized gradient approximation PBE functional and Grimme's semiempirical dispersion correction method D3 (BJ).^37–39^ A plane wave (PW) basis set with a cut-off energy of 400 eV was used in combination with the projected augmented wave (PAW)^40^ method. The Brillouin zone sampling was restricted to the Γ point. The convergence was assumed to be reached when the forces on the atoms were below 0.05 eV Å^−1^. The ZSM-5 unit cell was optimized, and the following lattice parameters were used: *a* = 20.24, *b* = 20.01 and *c* = 13.44 Å. A supercell of MOR was constructed by a doubling monoclinic primitive cell along the *c* axis and with the lattice parameters of *a* = *b* = 13.65, *c* = 15.02 Å and *α* = *β* = 90.0° and *γ* = 97.2°. Bulk α-Al_2_O_3_ with optimized unit cell parameters as *a* = *b* = 7.72, *c* = 12.96 and *α* = *β* = 90.0° and *γ* = 120.0° was modelled to be used as the reference state for the EFAl aiTA calculations. The Brillouin zone sampling was performed using a 3 × 3 × 1 mesh. For comparison, the thermodynamic analysis involving a less stable bulk Al(OH)_3_ phase as a reference was carried out with the results summarized in Section S.3. of the ESI.†

### *Operando* model

The relative stabilities of the EFAl species in zeolite pores under the catalytically relevant conditions were next assessed in the framework of the aiTA method.^41–43^ The following equilibrium was considered for the stability assessment:E1
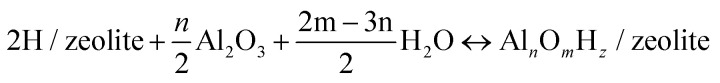


The reaction free energy is defined asE2
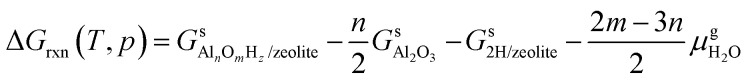
where 
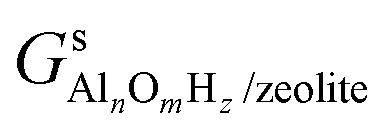
, 
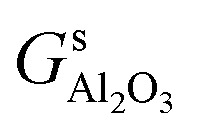
 and *G*^s^_2H/zeolite_ are the Gibbs free energies of the EFAl-containing zeolite model, bulk Al_2_O_3_ and parent EFAl-free acidic zeolite matrix (2H/zeolite). The bulk, 2H/zeolite and Al_*n*_O_*m*_H_*z*_/zeolite are the DFT-approximated energies of the aluminum oxide, the respective zeolite framework with two hydrogens and the frameworks with situated EFAls. The PV-contribution entropy of the solids could be neglected and the expression for the Gibbs free energy can be written as follows:E3
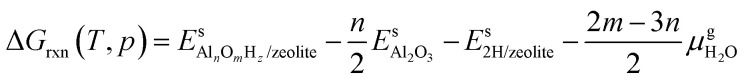
where 
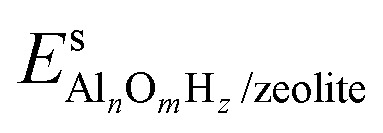
 and *E*^s^_2H/zeolite_ are the pDFT-energies of the given EFAl–zeolite and the parent H–zeolite structures, respectively. The condition dependencies of the reaction free energy are explicitly accounted for with 
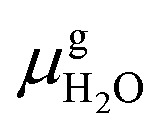
 defined asE4
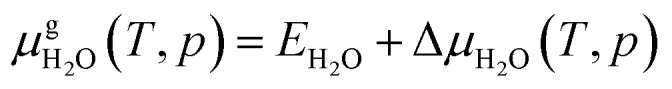
E5
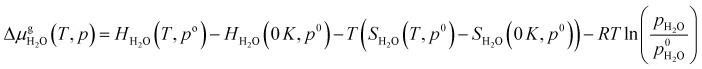


Thermodynamic tables were used to calculate the values of entropy and enthalpy at a standard pressure (1 bar) and different temperatures.^44^ Incorporating eqn (E4) and (E5) in eqn (E3) gives the change in the Gibbs free energy of the formation of the EFAl stoichiometries. This methodology has been successfully employed earlier on a wide range of solid systems including zeolite catalysts.^12,41,45^

## 3. Results and discussion

**Fig. 2:**
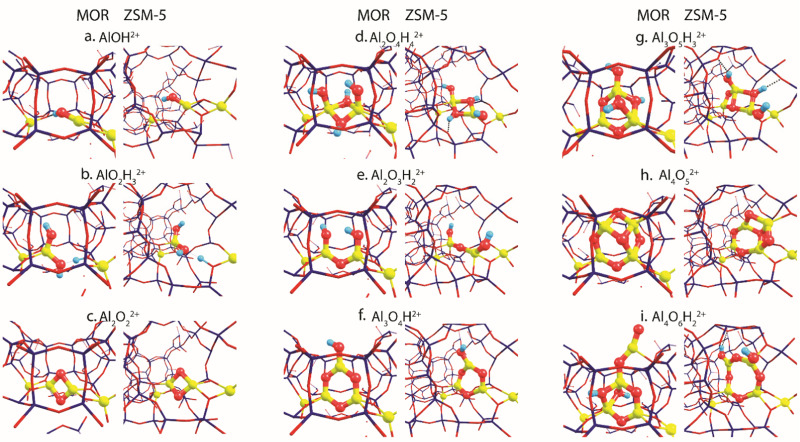
The global minima structures of various EFAl stoichiometries produced by GA runs and subsequently optimized in the MOR and ZSM-5 zeolite frameworks.

Fig. 2 summarizes the most stable EFAl geometries among the five DFT-optimized configurations (Fig. S5 and S6, ESI†) that were initially extracted from the bias-free *x*TB-based GA configurational search (Fig. S3 and S4, ESI†). The pDFT refinement of the *x*TB semi-empirically optimized structural isomers results in their substantial geometrical alterations. The presence of the artefacts in *x*TB predictions has been previously shown by Vicent-Luna in a form of undesirable structural distortions of geometries with lower symmetry after the optimization.^46^ This could be attributed to the key approximation within GFN1 representing the noncovalent interactions *via* the atom pair-wise electrostatic interactions described as spherically symmetric and monopole-types.^47^ Nevertheless, we found that the *x*TB-driven GA search provides a robust and efficient way to explore the configurational space of the intrazeolite extraframework species to obtain a good structural guess for the subsequent pDFT refinement. In the following, we predominantly discuss the pDFT refined data, while the *x*TB-derived predecessor structures will only briefly be discussed where appropriate.

The global minima geometries summarized in Fig. 2 indicate that, apart from the little distortions in the coordinational environment caused by the formation of the hydrogen bonds, similar EFAl geometries are found for particular stoichiometries in MOR and ZSM-5 zeolites. This stands for a majority of stoichiometries, except for the multinuclear Al_3_O_5_H_3_ and Al_4_O_6_H_2_ complexes, where structurally distinct features are promoted by the different confinement spaces.

For the smallest AlOH stoichiometry, a single stable EFAl type is revealed by pDFT represented by an AlOH^2+^ cation coordinated directly to the lattice basic oxygen ions of one of the framework AlO_2_^−^ anions with the second one providing the indirect charge-compensation of the cationic EFAl (Fig. 2a). The GA structural exploration has also revealed alternative configurations for the mordenite-confined AlOH represented by Al=O^+^ and an adjacent Brønsted acid site (Fig. S3c, ESI†), additionally stabilized within the narrower confinement space of the MOR side-pocket. Such configurations have been discussed in the previous EFAl literature.^19,48^ However, they were found unstable or underwent restructuring into the charge-alternative states during the pDFT refinement. Similarly, a single stable configuration is found for another mononuclear stoichiometry – AlO_2_H_3_, represented by a tetrahedral Al(OH)_2_^+^ EFAl cation coordinated to one lattice AlO_2_^−^ anion with the second one charge-compensated by the H^+^ BAS site (Fig. 2b).

Binuclear stoichiometries include Al_2_O_2_ and more hydrated/hydroxylated complexes such as Al_2_O_4_H_4_ and Al_2_O_3_H_2_. For both zeolites, the former species adopts a symmetrical diamond-shape binuclear Al(μ-O)_2_Al^2+^ configuration (Fig. 2c). This geometry is described as the most stable among all DFT-optimized structures, whereas some of their *x*TB-optimized predecessors reveal the presence of a linear isomer OAl(μ-O)Al which has one bridging oxygen and one terminal oxygen (Fig. S4a, ESI†), similar to the structure proposed earlier for the EF–Ga species.^49^ In Fig. 2d and e, Al_2_O_4_H_4_ and Al_2_O_3_H_2_ geometries respectively form symmetrical complexes that have minor distinctions in the hydrogen bond arrangements and proximity to the Al framework sites, affecting their effectivity of compensating the negative charge of the framework.

More complex trinuclear species such as Al_3_O_4_H and Al_3_O_5_H_3_ display more possibilities of structural arrangements. In Fig. 2f, Al_3_O_4_H stoichiometries exhibit the formation of a 6-membered ring with a coordinated OH-group, which is slightly distorted in ZSM-5 due to the formation of the hydrogen bonds between the OH group and the framework. The optimization of the A_3_O_5_H_3_ complexes gives a ring-shaped Al_3_O_3_H core moiety in ZSM-5 and an envelope-like Al_3_O_3_H core structure in MOR, which also coordinates two additional OH-groups of the structure. (Fig. 2g).

Fig. 2h and i show the EFAl moieties with the highest number of aluminum atoms considered in this study. A more hydrated Al_4_O_6_H_2_ (2i) species in mordenite accommodates a ring-shaped Al_3_O_3_H moiety with the bound OH-group at the same time expelling AlO_2_ species out of the pocket. Its ZSM-5 counterpart, on the other hand, could accommodate an 8-membered EFAl ring with two coordinated OH-groups. Another stable configuration of Al_4_O_6_H_2_ is a 6-membered EFAl with the OH-group and expelled AlO_2_H species as shown in Fig. S6i of the ESI.† For the Al_4_O_5_ stoichiometry in Fig. 2h, the most stable isomers adapt envelope-like Al_3_O_3_ geometries linked to an AlO_2_ moiety species in both zeolite frameworks. However, a more structurally diverse picture could be seen in a detailed 5-structure analysis. In Fig. S5h (ESI†), the Al_4_O_5_ geometry, when hosted in mordenite, tends to adapt in some cases coordination resembling the one from Al_4_O_6_H_2_ with an AlO_2_ species dislodged from the side pocket. The observed accommodation of the smaller species in the side pocket of MOR than that in ZSM-5 can be explained by a stronger confinement and limited pore size in mordenite.

**Fig. 3:**
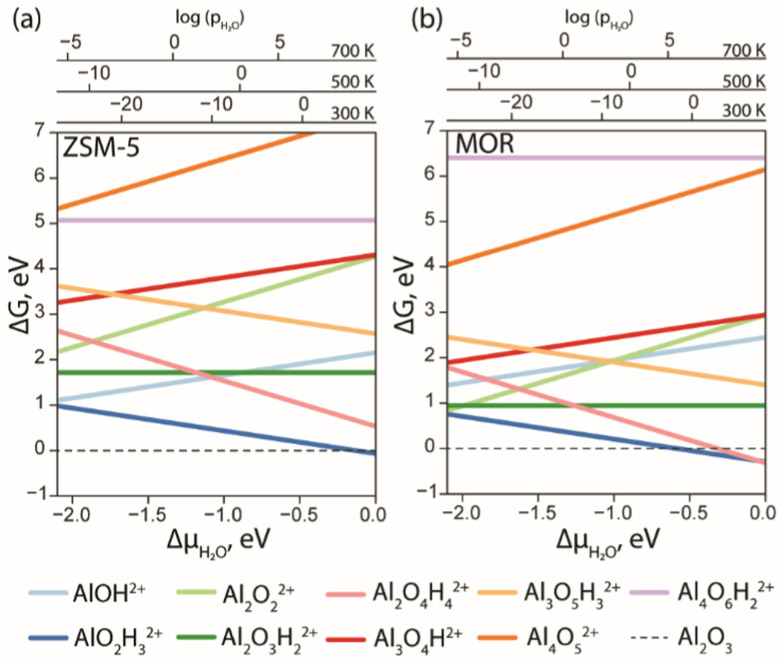
The Gibbs free energy diagram of the EFAl species confined in ZSM-5 (a) and MOR (b) was calculated with respect to the α-Al_2_O_3_ phase as a function of water chemical potential.

With the results of DFT optimization being sufficiently elaborated, the respective stabilities of the structures under *operando* conditions could be compared. In Fig. 3, the *ab initio* thermodynamics analysis shows the Gibbs free energies of the formation of different stoichiometries as a function of water chemical potential (Δ*μ*_H_2_O_). The values of the Gibbs free energies are computed using eqn (E3) and the Δ*μ*_H_2_O_ is derived from E5. The logarithmic pressure scales above the diagram represent the pressures required for the high (700 K and 500 K) and moderate (300 K) temperature processes.

The computed results reveal a general trend of the thermodynamic preference for the formation of mononuclear and binuclear cationic EFAl species in both zeolite topologies at high water chemical potentials, which is in line with the experimental findings.^16,19,28^ At low water chemical potential, the formation of a separate bulk aluminum oxide and zeolite BAS is predicted for the current highly stable α-Al_2_O_3_ reference structure. The cationic EFAl species are better stabilized when confined within the mordenite side pocket, as is evident from generally lower relative free energies predicted for all EFAl stoichiometries compared to those in the MFI. The largest cation Al_4_O_6_H_2_^2+^ deviates from this trend, which because of its size cannot be accommodated within the narrow MOR side pocket resulting in the expulsion of quite an unstable O–Al=O moiety into the main channel.

The thermodynamics of EFAl speciation is highly condition-dependent. At low chemical water potentials (*μ*_H_2_O_ < −1.0 eV), the formation of bulk α-Al_2_O_3_ oxide is preferred over the molecular cationic EFAl species indicating the limited possibility of the redispersion of Al upon the calcination zeolite activation procedures. Nevertheless, in the MOR zeolite, AlO_2_H_3_, Al_2_O_3_H_2_, Al_2_O_2_ and Al_2_O_4_H_4_ EFAl species can be found within 1 eV from the reference value, whereas they are an additional 1 eV less stable in ZSM-5. The mononuclear AlO_2_H_3_ and binuclear Al_2_O_4_H_4_ species are thermodynamically favoured at high *μ*_H_2_O_, indicating that such isolated cationic sites can potentially be formed upon *e.g.* steaming or ion exchange and further stabilized kinetically in the activated material. The formation of EFAl species with a higher degree of nuclearity (three- and four-nuclear) is strongly thermodynamically unfavourable under all conditions, which is in sharp contrast to the situation previously discussed for low-silica faujasite, where trinuclear multiple-charged EFAl confined in small sodalite cages were among the most stable species.^16^

The predicted EFAl stabilities depend on the specific choice of the bulk Al reference phase. We have also considered a fully hydrated Al(OH)_3_ as a bulk reference state in aiTA (see Fig. S7 in Section S.3. of the ESI†). Because of the hydrated nature of the reference structure, the less hydrated multinuclear aggregates appear more stable at low *μ*_H_2_O_ in this case. Nevertheless, the preferential formation of mono- and binuclear cationic species at higher *μ*_H_2_O_ values relevant to the conditions of steam-calcination remains unchanged by the choice of the bulk Al reference.

Our results point to a preference for the increased hydration of EFAl at higher *μ*_H_2_O_; however, we should also emphasize that the higher degrees of hydration could destabilize the EFAl/zeolite systems as water holds the potential to cleave Al–O–(H)–bonds,^50^ which could potentially manifest itself for stoichiometries with formal H_2_O contents above those considered in this study. The outcome of the GA search and the following aiTA procedure vividly indicated that a smaller pore size in mordenite offers a tighter confinement effect and better stabilization of the cationic species up to a certain size. The relative stability of the EFAl species when compared to the α-Al_2_O_3_ reference phase indicated a more pronounced stabilization of the mononuclear and binuclear species and the destabilization of the larger agglomerates due to the localized and disperse nature of the lattice negative charge and unfavourable confinement in MOR and ZSM-5. This explains the differences in EFAl speciation predicted for low-silica cage-type faujasite zeolites and suggests a different promoting effect of these species in the different zeolite topologies.

## Conclusions

In this study, we applied the semi-empirical-based GA to investigate the structural uncertainty surrounding the EFAl species confined within zeolites. The catalytic importance of these extraframework species motivated us to research the configurations of different EFAl nuclearities in two zeolitic frameworks such as MOR and ZSM-5. Varying the stoichiometry of the EFAl accounts for the different concentrations of aluminum. For this purpose, the number of aluminum atoms ranging from 1 to 4 was considered in our study. Different zeolite topologies impose a distinct confinement effect on the anchored extraframework species. Here, we have shown that, within a chosen level of theory, the *x*TB-based GA method is effective for the structural exploration of complex EFAl species in zeolites by considering 9 different stoichiometries in MOR and ZSM-5. However, the structural and energy refinement at the DFT level is necessary to form a basis for the accurate and reliable structural evaluation and stability analysis.

The low-nuclearity EFAl structures are coherent with the findings reported by a previous study, thereby validating the proposed computational workflow. In addition, more complex multinuclear EFAl configurations were investigated and included in the stability analysis. To analyse the EFAl stability under *operando* catalytically relevant conditions, the relative energetics of the pDFT-refined low-lying structures was estimated by embedding them in the *ab initio* thermodynamics analysis. It was shown that both ZSM-5 and MOR topologies favour the formation of the cationic mononuclear EFAl in the presence of water/water vapors. Specifically, the vicinal Brønsted acid site and mononuclear Al(OH)_2_^+^ cation representing the AlO_2_H_3_ EFAl stoichiometry were found to be the most thermodynamically stable at high *μ*_H_2_O_ in both zeolite frameworks. However, at low water chemical potential values, the formation of bulk Al_2_O_3_ is more thermodynamically favourable. This can be attributed to the restricted confinement space in MOR and ZSM-5 channels. Our calculations suggest a generally better stabilization of a majority of the EFAl species in the MOR zeolite.

Thus, we show that GA-based global optimization in combination with pDFT structural refinement and *ab initio* thermodynamics analysis provides an efficient computational workflow for the expert-bias-free structural prediction on intrazeolite extraframework ensembles and evaluating their stabilities under catalytically relevant conditions. By using the catalytically important EFAl species embedded in MOR and ZSM-5 frameworks, we demonstrate that our proposed approach can be a powerful tool to address the structural conundrum of extraframework speciation in zeolite-based catalysts.

## Author contributions

The manuscript was written *via* contributions from all the authors. All the authors have approved the final version of the manuscript.

## Conflicts of interest

There are no conflicts to declare.

## Supplementary Material

CP-024-D2CP03603G-s001

CP-024-D2CP03603G-s002
