# Supplementary material for: Global optimization of extraframework ensembles in zeolites: structural analysis of extraframework aluminum species in MOR and MFI zeolites
Source: Phys Chem Chem Phys. 2022 Oct 11;24(44):27047–54. doi: 10.1039/d2cp03603g (PMC9673684; doi:10.1039/d2cp03603g)
Supplement: CP-024-D2CP03603G-s001 [file CP-024-D2CP03603G-s001.pdf]

Electronic Supplementary Material (ESI) for Physical Chemistry Chemical Physics.
This journal is © the Owner Societies 2022

SUPPORTING INFORMATION

Global optimization of extraframework ensembles in zeolites: structural analysis of extraframework aluminum species in MOR and MFI zeolites

Elena V. Khramenkova,[a,‡] Harshini Venkatraman,[a,‡] Victor Soethout[a] and Evgeny A. Pidko[a*]

[a] Inorganic Systems Engineering, Department of Chemical Engineering

Delft University of Technology

Van der Maasweg 9, 2629 HZ Delft, The Netherlands

*: Evgeny A. Pidko, e.a.pidko@tudelft.nl

S1. The topologies of the MOR and ZSM-5 frameworks.

Different zeolites' topologies have different arrangements and size pores influencing the accessibility of the extraframework species and the proximity of the BAS.[1,2] Therefore, it is important to evaluate the structural features of each topology used in this study. A structure of a MOR has a one-dimensional pore system including the main channel with a diameter of 6.7 x 7.0 Å and a compressed channel with a diameter of 2.6 x 5.7 Å. It was found to be efficient in catalyzing cracking, hydroisomerization, hydrocracking and alkylation.[3] As a one-dimensional zeolite, it might experience diffusion constraints and deactivation by pore blockage. A structure of a ZSM-5 framework has an MFI topology, with intersecting straight and sinusoidal 10-membered ring channels, with a channel of dimeter 5.1 x 5.5 Å. This topology is extensively employed in synthesizing hydrocarbons from methanol (MTH), nitrous oxide decomposition and oxidation of benzene to phenol.[4]

S2. The choice of the portion of the low-energy structures extracted from GA for the DFT refinement.

To define the required portion of the GA population pool for finding the low-energy structure, we have performed a test study on the $[AlOH]^{2+}$ and $[AlO_2H_3]^{2+}$ stoichiometries, where 9 and 17 structures from the population pool were used respectively. The lowest-lying cluster structures obtained from the GA search were optimized at the PBE-D3(BJ) level of theory to evaluate if the increase in theory level causes changes in energy trends and geometries.[5–8] These cluster DFT calculations were carried out using CP2K software package with the orbital transformation and Quickstep module for faster convergence.[9] The Goedecker-Teter-Hutter pseudopotentials and DZVP-MOLOPT-SR-GTH basis sets were used.[10,11] The changes in the relative electronic

energies when the level of the theory increases are summarized in Figures S1 and S2. For the $[AlOH]^{2+}$ stoichiometry, the global minimum observed in the GA search forms AlO^+ and H^+ species, which are both bound to different framework Al sites (structure b in Figure S1). The other low-lying structure observed (structure a in Figure S1) has the $[AlOH]^{2+}$ site which is bound to a single framework Al embedded in the zeolite. Figure S1 shows that within the 5 lowest-lying configurations found in the GA search, the global minimum can be found which is supported by the DFT-level optimization.

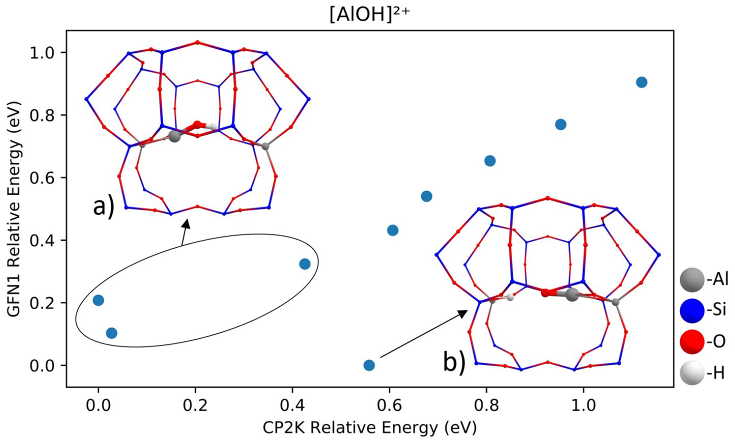

Figure S1. The test study assesses the correlation between the electronic energies of the lowest-lying structures obtained from the xTB-based GA search and the electronic energies obtained from the following single-point DFT-based calculation. The comparison was conducted on $[AlOH]^{2+}$ cluster configurations confined in the side pocket of mordenite. The level of theory employed for the xTB is GFN1, while the DFT calculations were carried out at the PBE-D3(BJ) level of theory.

Another stoichiometry – $[AlO_2H_3]^{2+}$ – has shown general agreement between the *x*TB and PBE calculations. The best correlation has been achieved for the formation of the $[AlO_2H_2]^+$ species over one framework Al site and H^+ over the other (structures c and d in Figure S2). As for the other isomer, $[AlO_2H_3]^{2+}$ site, which is bound to a single framework Al site, higher stability was predicted in the PBE-level optimization. Therefore, based on the inconsistencies between the energies assigned by the *x*TB and DFT methods to the same global minimum defined in the GA

search, we have considered a portion of the low-energy structures from the population pool instead of a single global minimum structure. The choice was made for the 5 lowest structures to capture the electronic deviations caused by the semi-empirical *x*TB method at a reasonable computational cost.

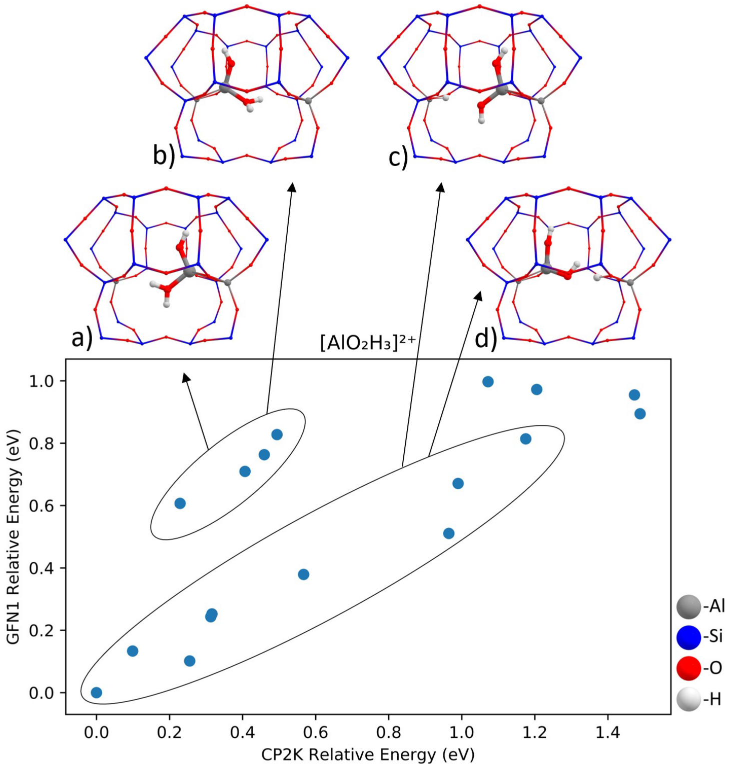

Figure S2. The test study assesses the correlation between the electronic energies of the lowest-lying structures obtained from the *x*TB-based GA search and the electronic energies obtained from the following single-point DFT-based calculation. The comparison was conducted on $[AlO_2H_3]^{2+}$ cluster configurations confined in the side pocket of mordenite. The level of theory employed for the *x*TB is GFN1, while the DFT calculations were carried out at the PBE-D3(BJ) level of theory.

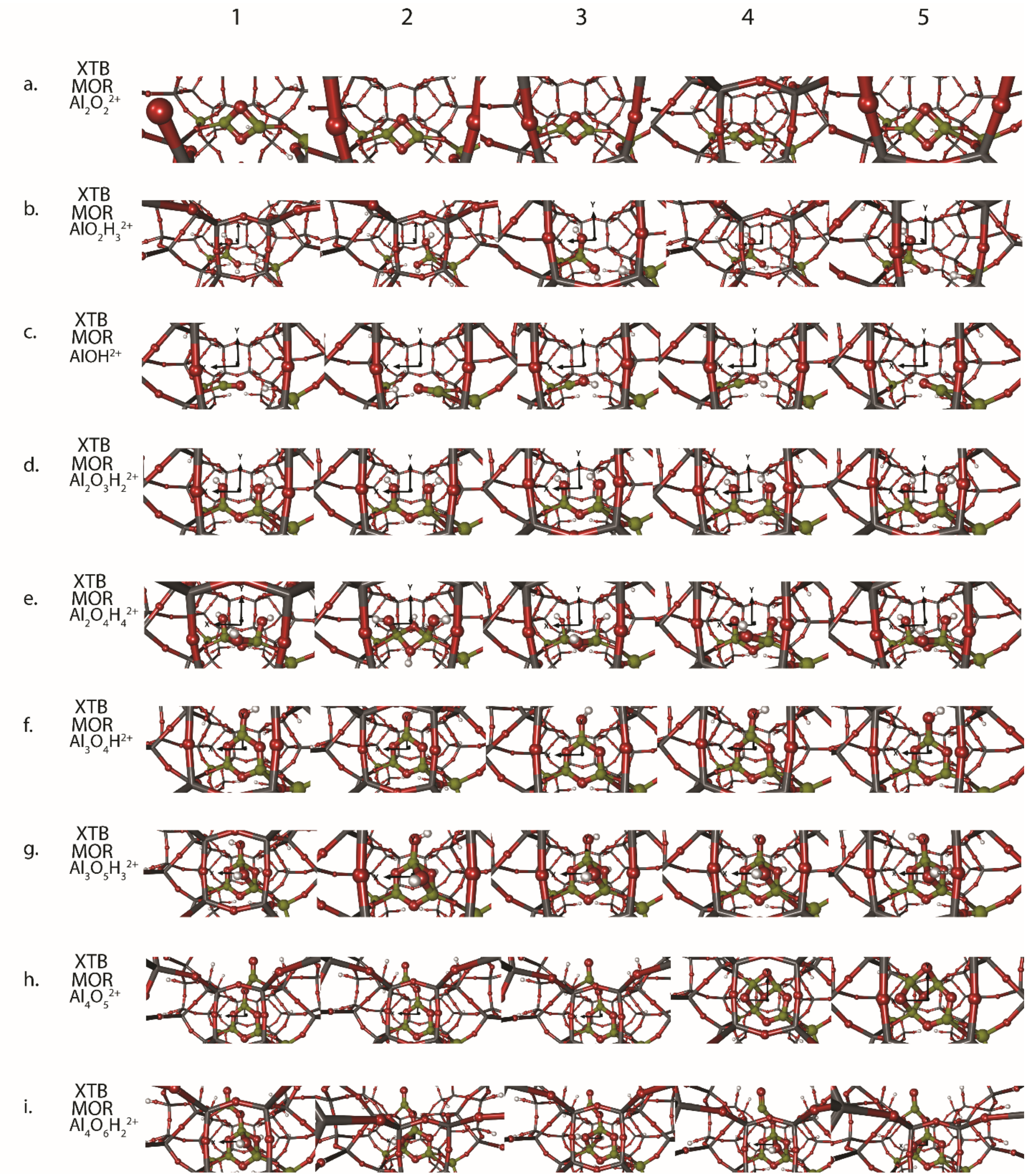

Figure S3. The five lowest-lying geometries for each stoichiometry confined in the side pocket of MOR generated in the final population pool of the GA procedure.

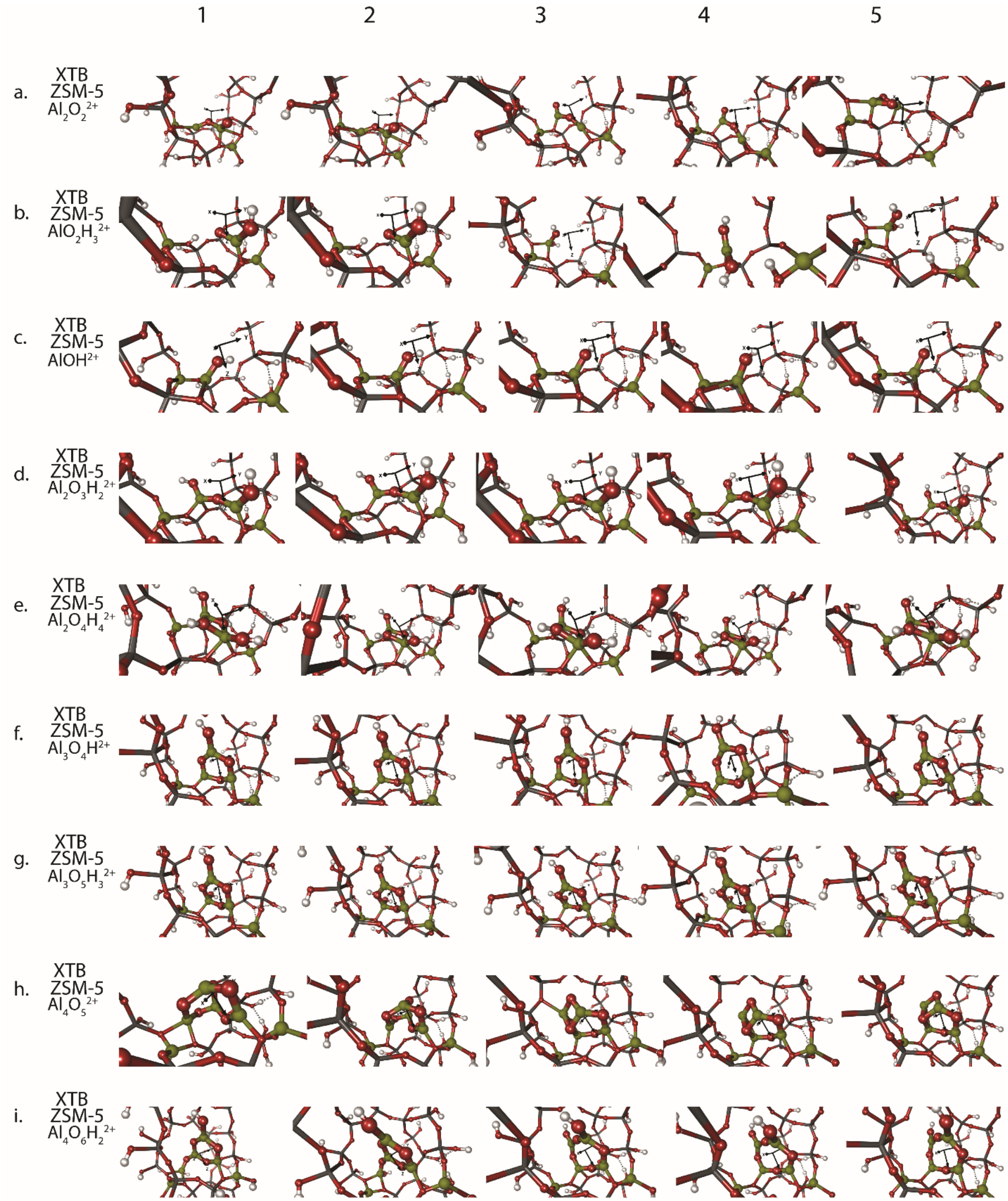

Figure S4. The five lowest-lying geometries for each stoichiometry confined in the gamma site of ZSM-5 generated in the final population pool of the GA procedure.

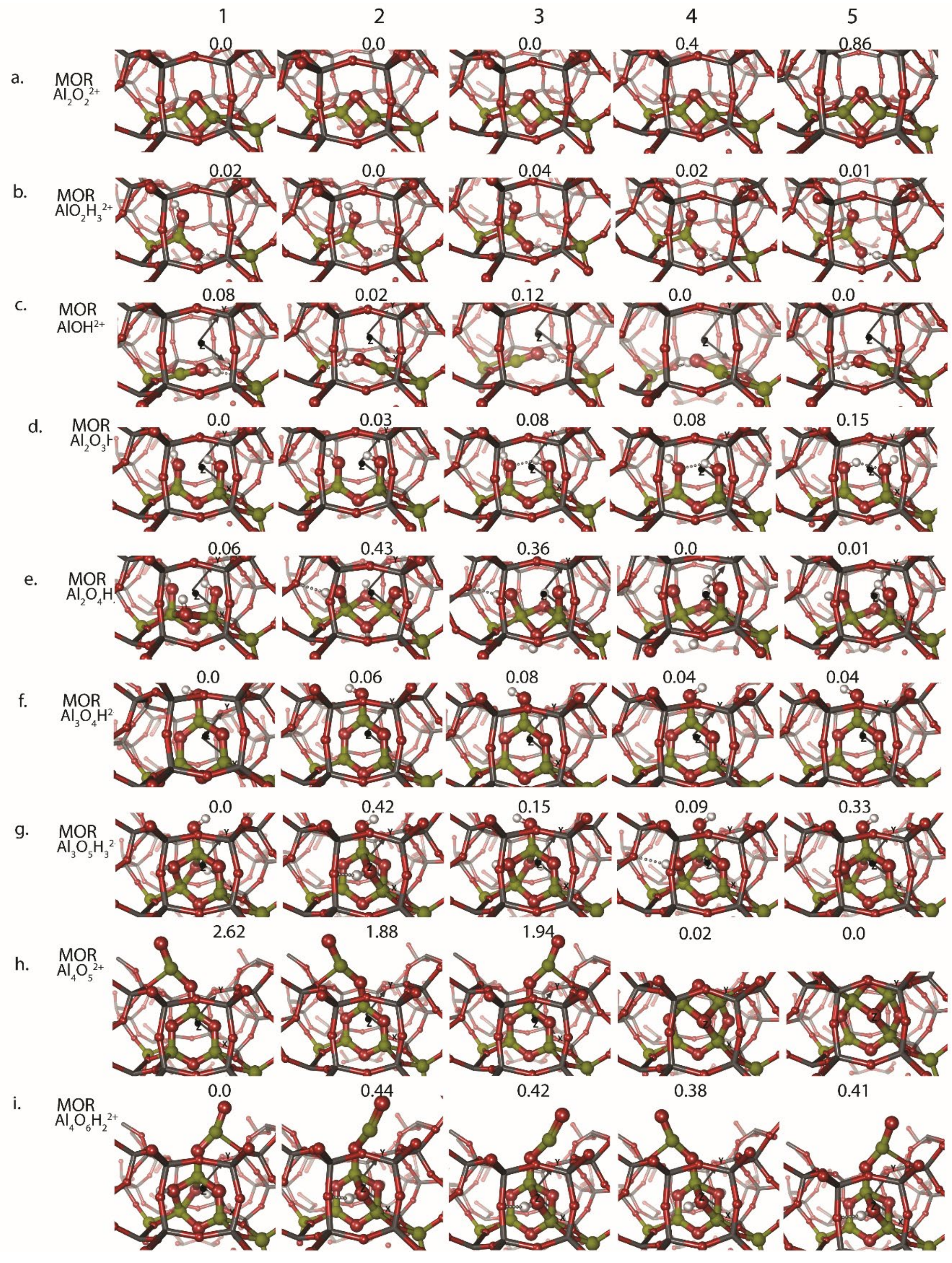

Figure S5. The five lowest-lying geometries for each stoichiometry confined in the side pocket of MOR obtained from the GA final population pool and optimized in the periodic cell at the PBE-D3(BJ) level of theory. The energies are on the top of the structures in eV with respect to the lowest-lying configuration in a row.

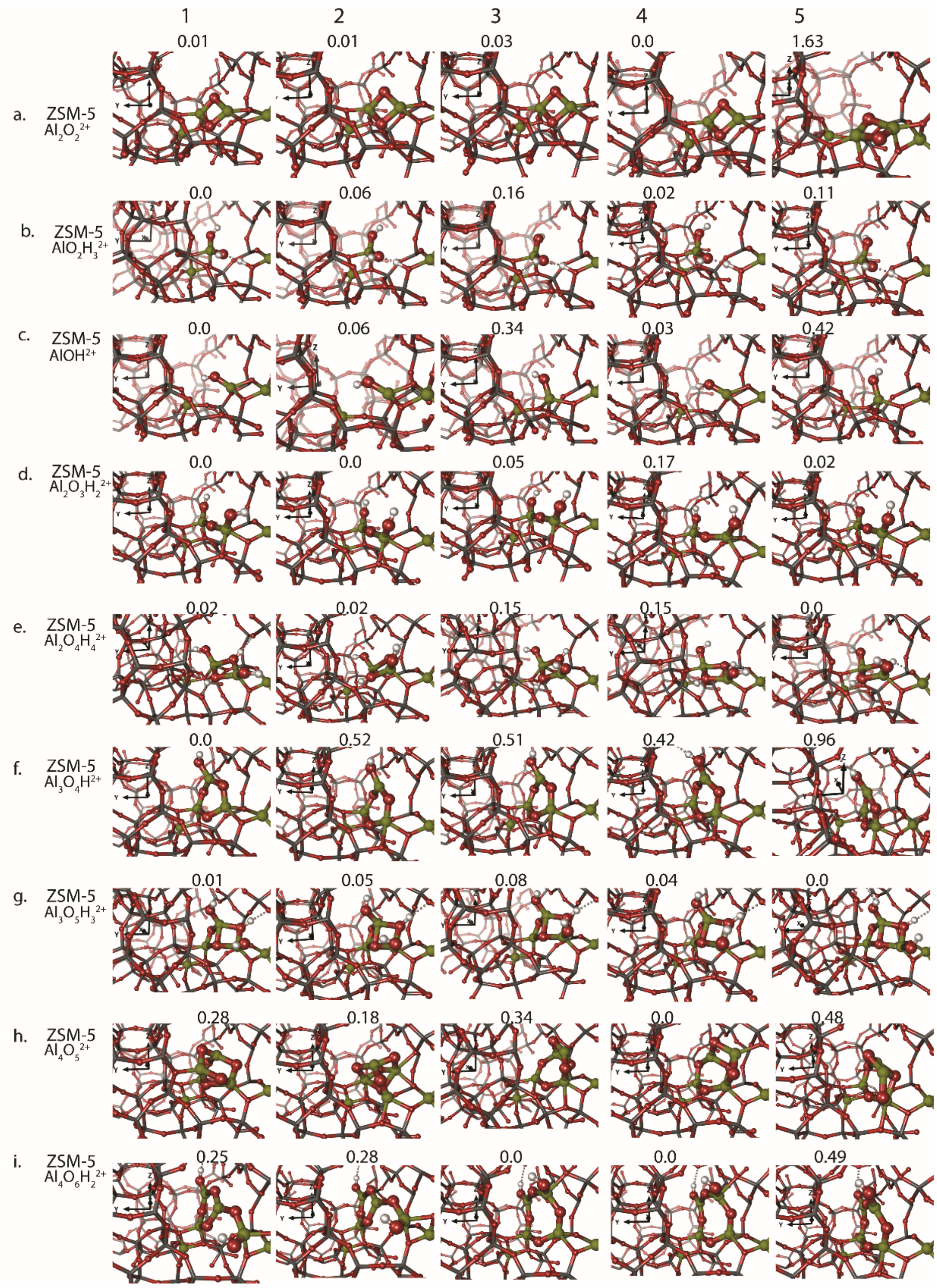

Figure S6. The five lowest-lying geometries for each stoichiometry confined in the gamma site of ZSM-5 obtained from the GA final population pool and optimized in the periodic cell at the PBE-D3(BJ) level of theory. The energies are on the top of the structures in eV with respect to the lowest-lying configuration in a row.

S3. Supplementary aiTD analysis with $Al(OH)_3$ as a reference system.

The dealumination treatments in zeolites can lead to the formation of abundant EFAl species. To account for an alternative source of EFAl in the system – $Al(OH)_3$ – another aiTA diagram has been constructed employing $Al(OH)_3$ as a reference structure. The following equilibrium was considered for the stability assessment:

$$2H/zeolite + n \cdot Al(OH)_3 + \frac{z-2-3n}{2} \cdot H_2O \leftrightarrow Al_nO_mH_z/zeolite \quad \text{SE.1}$$

The reaction free energy is defined as:

$$\Delta G_{rxn}(T,p) = G^{s}_{Al_nO_mH_z/zeolite} - n \cdot G^{s}_{Al(OH)_3} - G^{s}_{2H/zeolite} - \frac{z-2-3n}{2} \cdot \mu^{g}_{H_2O} \quad \text{SE.2}$$

where, $G^{s}_{Al_nO_mH_z/zeolite}$, $G^{s}_{Al(OH)_3}$ and $G^{s}_{2H/zeolite}$ are the Gibbs free energies of the EFAl-containing zeolite model, bulk $Al(OH)_3$ and parent EFAl-free acidic zeolite matrix (2H/zeolite). The bulk, 2H/zeolite and $Al_nO_mH_z$/zeolite are the DFT-approximated energies of the gibbsite, the respective zeolite framework with two hydrogens and the frameworks with situated EFAls. The PV-contributions entropy of the solids could be neglected and the expression for the Gibbs free energy can be written as:

$$\Delta G_{rxn}(T,p) = E^{s}_{Al_nO_mH_z/zeolite} - n \cdot E^{s}_{Al(OH)_3} - E^{s}_{2H/zeolite} - \frac{z-2-3n}{2} \cdot \mu^{g}_{H_2O} \quad \text{SE.3}$$

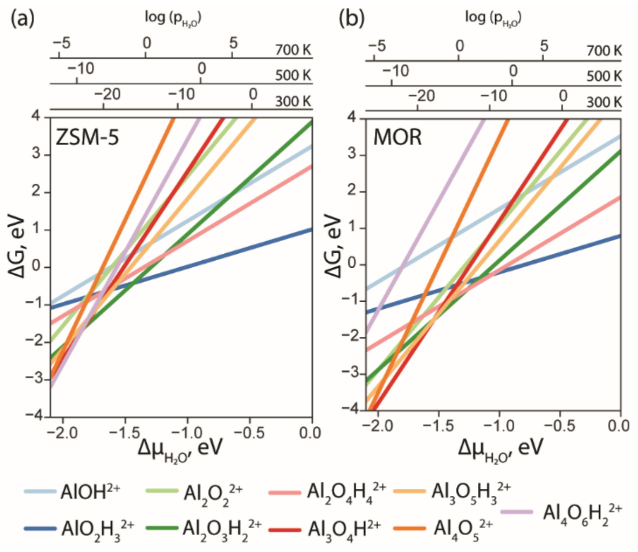

Figure S7. The Gibbs free energy diagram of the EFAl species confined in ZSM-5 (a) and in MOR (b) was calculated with respect to the $Al(OH)_3$ phase as a function of water chemical potential.

References

1 W. Xu, S. J. Miller, P. K. Agrawal and C. W. Jones, Zeolite topology effects in the alkylation of phenol with propylene, *Appl. Catal. A Gen.*, 2013, **459**, 114–120.

2 W. N. P. van der Graaff, C. H. L. Tempelman, E. A. Pidko and E. J. M. Hensen, Influence of pore topology on synthesis and reactivity of Sn-modified zeolite catalysts for carbohydrate conversions, *Catal. Sci. Technol.*, 2017, **7**, 3151–3162.

3 J. Aguado, D. P. Serrano, J. M. Escola and A. Peral, Catalytic cracking of polyethylene over zeolite mordenite with enhanced textural properties, *J. Anal. Appl. Pyrolysis*, 2009, **85**, 352–358.

4 R. Joyner and M. Stockenhuber, Preparation, Characterization, and Performance of Fe−ZSM-5 Catalysts, *J. Phys. Chem. B*, 1999, **103**, 5963–5976.

5 J. P. Perdew, K. Burke and M. Ernzerhof, Generalized gradient approximation made simple, *Phys. Rev. Lett.*, 1996, **77**, 3865–3868.

6 S. Grimme, J. Antony, S. Ehrlich and H. Krieg, A consistent and accurate ab initio parametrization of density functional dispersion correction (DFT-D) for the 94 elements H-Pu, *J. Chem. Phys.*, , DOI:10.1063/1.3382344.

7 S. Grimme, S. Ehrlich and L. Goerigk, Effect of the damping function in dispersion corrected density functional theory, *J. Comput. Chem.*, 2011, **32**, 1456–1465.

8 J. Vandevondele, M. Krack, F. Mohamed, M. Parrinello, T. Chassaing and J. Hutter, Quickstep: Fast and accurate density functional calculations using a mixed Gaussian and plane waves approach, *Comput. Phys. Commun.*, 2005, **167**, 103–128.

9 T. D. Kühne, M. Iannuzzi, M. Del Ben, V. V Rybkin, P. Seewald, F. Stein, T. Laino, R. Z. Khaliullin, O. Schütt and F. Schiffmann, CP2K: An electronic structure and molecular dynamics software package-Quickstep: Efficient and accurate electronic structure calculations, *J. Chem. Phys.*, 2020, **152**, 194103.

10 J. VandeVondele and J. J. Hutter, Gaussian basis sets for accurate calculations on molecular systems in gas and condensed phases, *J. Chem. Phys.*, 2007, **127**, 114105.

11 M. Krack, Pseudopotentials for H to Kr optimized for gradient-corrected exchange-correlation functionals, *Theor. Chem. Acc.*, 2005, **114**, 145–152.
